# Honey dressing: a missed way for orthopaedic wound care

**DOI:** 10.1007/s00264-022-05540-9

**Published:** 2022-08-16

**Authors:** Abdel-Salam Abdel-Aleem Ahmed, Sherif Eltregy, Mahmoud Ibrahim Kandil

**Affiliations:** Department of Orthopaedic Surgery, Benha Faculty of Medicine, Benha University, Farid Nada Street, Kalyubia, Benha, Post Office 13518 Egypt

**Keywords:** Orthopaedic wounds, Open fractures, Infected non-union, Honey dressing, Postoperative infections, Ilizarov

## Abstract

**Purpose:**

Orthopaedic-related wounds are critical situations calling for care to avoid deep infections and its consequences. The purpose of this study was to evaluate the efficacy of using honey for care of orthopaedic-related wounds with limited resources.

**Patients and methods:**

This prospective study included 50 cases with an average age of 38.18 (range 17–63) years with 38 males and 12 females. The most frequent wound location was the leg (41 patients; 82%), then the foot (six patients; 12%), and the ankle in three patients (6%). The aetiologies were open fractures (34 cases; 68%), infected tibial non-unions (nine cases; 18%), and post-operative infections (seven cases; 14%). Exposed tendon was present in three cases. Bone exposure was present in two cases. Deep infection was present in 29 cases (58%). Besides treating the primary cause, a ribbon of gauze soaked with honey was applied to the wounds after thorough saline washing.

**Results:**

Wound sizes were variable. All cases showed improvement in all parameters with complete wound healing and full coverage of bone and tendons. Recurrence of deep infection occurred in three cases and treated by debridement. One case needed sequestrectomy of a small exposed tibial cortical fragment. Exposed tendon cases showed superficial necrosis which was treated by simple debridement. Initial mild itching occurred in five patients with spontaneous resolution.

**Conclusion:**

With treating the underlying aetiology and optimising the patient’s general condition, honey was an effective, simple, and affordable method of wound care in different orthopaedic conditions even with exposed bone or tendons.

## Introduction

Orthopaedic-related wounds are critical situations that necessitate a special care to avoid occurrence of infection and its debilitating influences on the life quality of the affected patients. Deep bone and joint infections are devastating complications which pose a formidable challenge confronted by the orthopaedic surgeons. Recognising the distinctive physiologic and anatomic characteristics of bone infections, prevention is the best course of action. Wound care is an important surgeon-dependent risk factor for infection besides prophylactic antibiotics, operating room environment and surgical technique [1].

Orthopaedic surgical dressings are classified into three categories. Passive dressings (as gauze, absorbent pads and adhesive tapes) act by physical wound protection and control of exudate. Active dressings (as films, hydrocolloids, hydrofiber and foam) provide a moist environment that promotes healing and their adherence to the wound is less likely. Interactive dressings (as antimicrobials, e.g. dressings containing silver or iodine, and vacuum dressings) augment the mechanisms of wound healing [2].

Honey is not only a high sugar-containing solution but also a biological wound dressing having many bioactive components which can enhance wound healing by several mechanisms [3]. Honey accelerates wound healing through actions on its three phases of inflammation, proliferation and remodelling. It has antioxidant and anti-inflammatory actions. This anti-inflammatory effect diminishes oedema and exudate and minimises or even prevents hypertrophic scar formation. It stimulates collagen synthesis, angiogenesis and granulation tissue formation, promotes epithelialisation, and enhances wound contraction. It reduces pain, deodorises the wounds and has a debriding action lifting the debris from the wound [4–6]. Moreover, the high viscosity of honey provides a protective barrier preventing infection [7]. Finally, yet importantly, honey has antimicrobial effects based on a multitude of factors diminishing the bioburden of wounds [3, 8, 9].

Honey had been used to treat wounds for thousands of years in multiple cultures. It was recorded in an Egyptian surgical text traced back to between 2600 and 2200 BCE [9, 10]. In the modern medicine, successful results have been achieved after honey application to many wounds, such as burns, different chronic ulcers, infected surgical wounds, malignant wounds, Fournier’s gangrene and neonatal wounds, along with others [4, 5, 8].

The studies reporting on the use of honey as method of treating orthopaedic-related wounds are few. The purpose of this prospective study was to evaluate the effectiveness and safety of using honey as topical care for orthopaedic-related wounds with limited resources.

## Patients and methods

This prospective study was conducted between January 2018 and April 2020 after the approval of the Research Ethics Committee at our Faculty of Medicine, University (REC-FOMBU). The inclusion criteria were patients having leg and foot wounds such as those secondary to open fractures, post-operative wound complications (infection and dehiscence) and infected tibial non-unions. Patients who refused honey as a method of wound care, cases with peripheral vascular insufficiency and cases that were missed during follow-up were excluded from the study. The study included 50 cases with an average age of 38.18 (range 17–63; SD 12.06) years with 38 males (76%) and 12 females (24%). Right side was affected in 29 cases (58%) and the left side in the remaining 21 cases (42%). The most frequent wound location was the leg in 41 patients (82%), followed by the foot in six patients (12%), then the ankle in three patients (6%) (Table 1).

**Table 1:** Location and causes of wounds

Location		Open fracture *n* (%)	Infected non-union *n* (%)	Postoperative *n* (%)		Total *n* (%)	Exposed bone		Exposed tendon	
				Internal fixation	Wound complication		Yes	No	Yes	No
Leg	Proximal	4 (8)	1 (2)	2 (4)	0 (0)	7 (14)	0	7	0	7
	Middle	16 (32)	2 (4)	0 (0)	0 (0)	18 (36)	1	17	1	17
	Distal	8 (16)	6 (12)	2 (4)	0 (0)	16 (32)	1	15	1	15
Ankle		0 (0)	0 (0)	0 (0)	3 (6)	3 (6)	0	3	1	2
Foot		6 (12)	0 (0)	0 (0)	0 (0)	6 (12)	0	6	0	6
Total n (%)		34 (68)	9 (18)	7 (14)		50 (100)	2	48	3	47

**Fig. 1:**
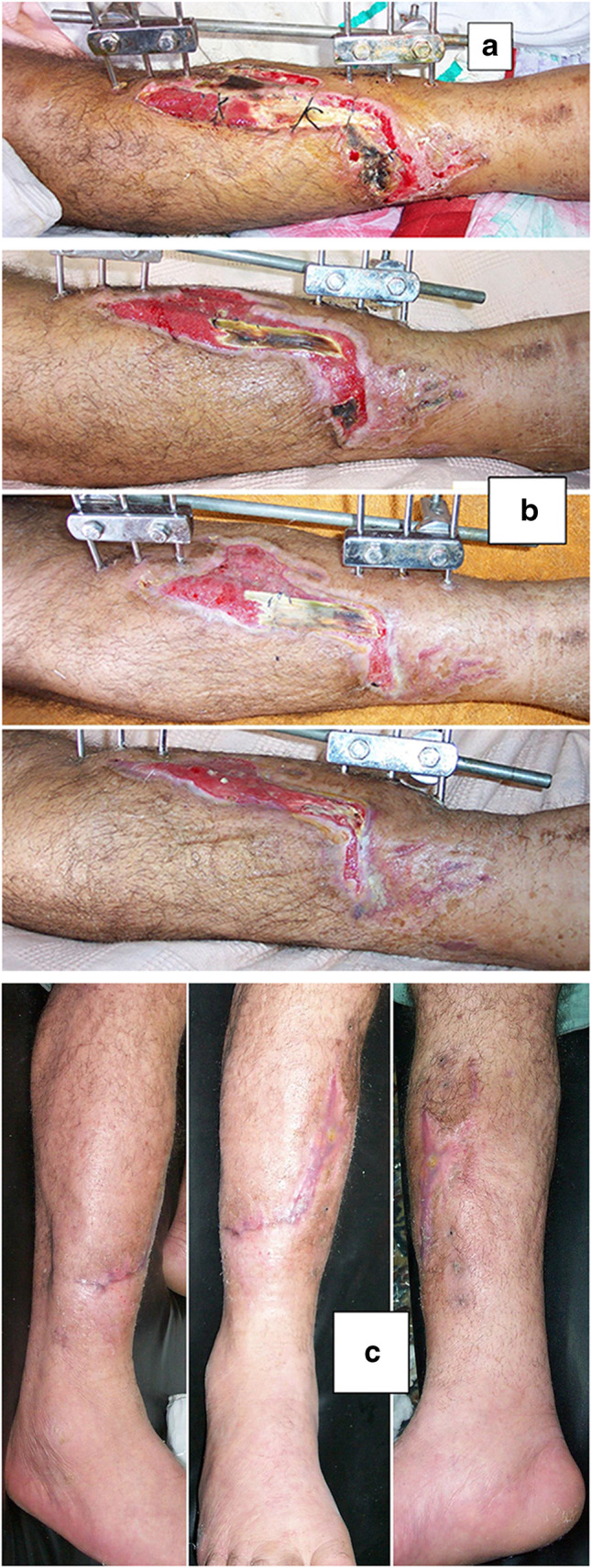
A 63-year-old male patient presented with grade III-A open tibial fracture treated by monolateral external fixator. **a** Wound condition with tendon exposure and skin sloughing after application of external fixator. **b** Clinical photos with of wound healing. **c** Clinical photos of sound wound healing

**Fig. 2:**
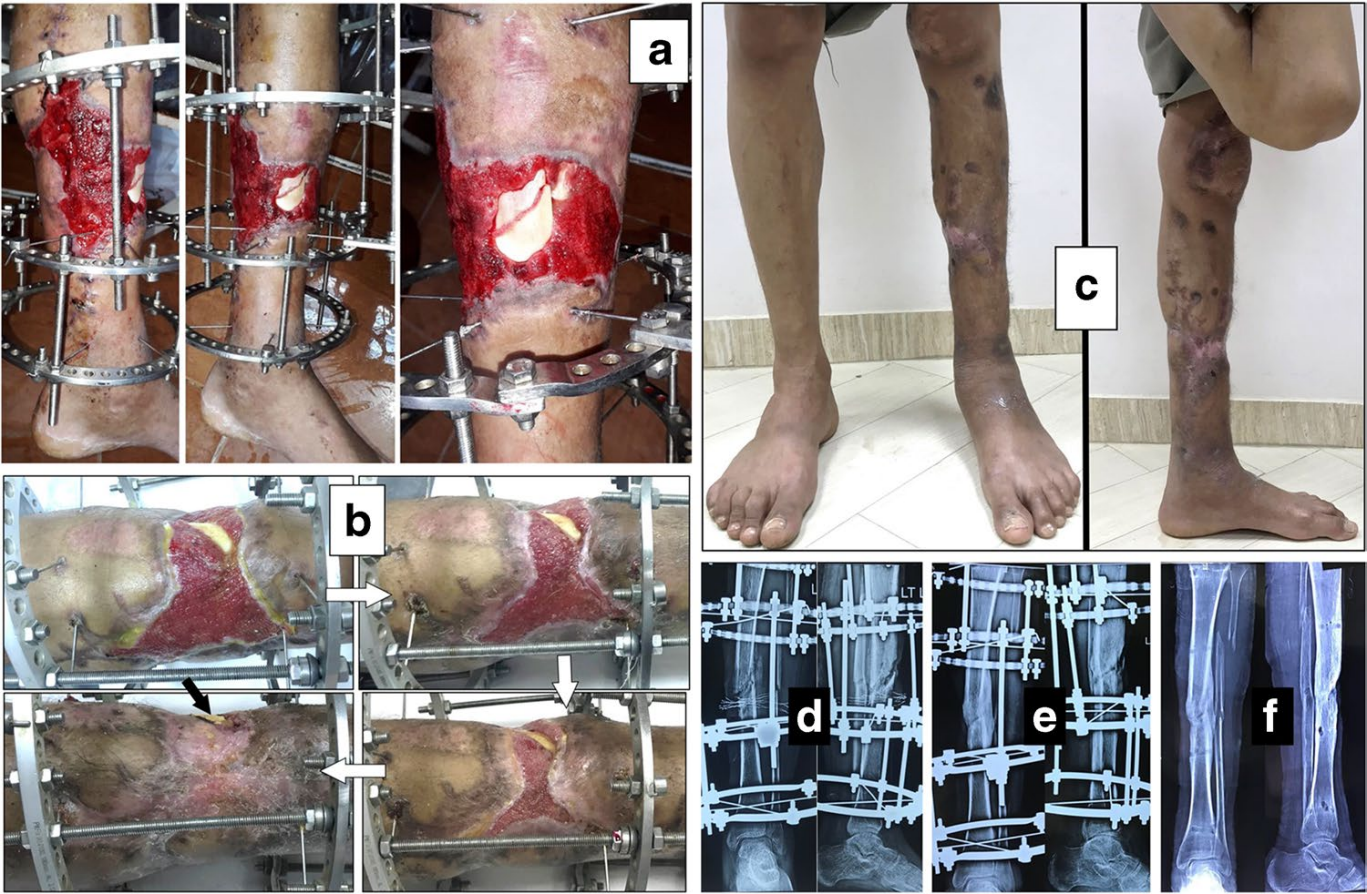
An 18-year-old smoker male patient presented after previous negative pressure wound therapy that was used to treat failed rotational flap in grade III-B open tibial fracture fixed by Ilizarov external fixator. **a** Different views of wound at presentation with bone exposure. **b** Clinical photos showing progression of wound repair following the white arrow. The black arrow points to a small anterior sequestered bone flake. **c** Clinical photos showing the standing patient with dry scar and complete bone coverage. **d**, **e**, **f** Radiographs at presentation, before Ilizarov frame removal with an anterior small sequestrum in the lateral view, and after fixator removal with sound bone union

**Fig. 3:**
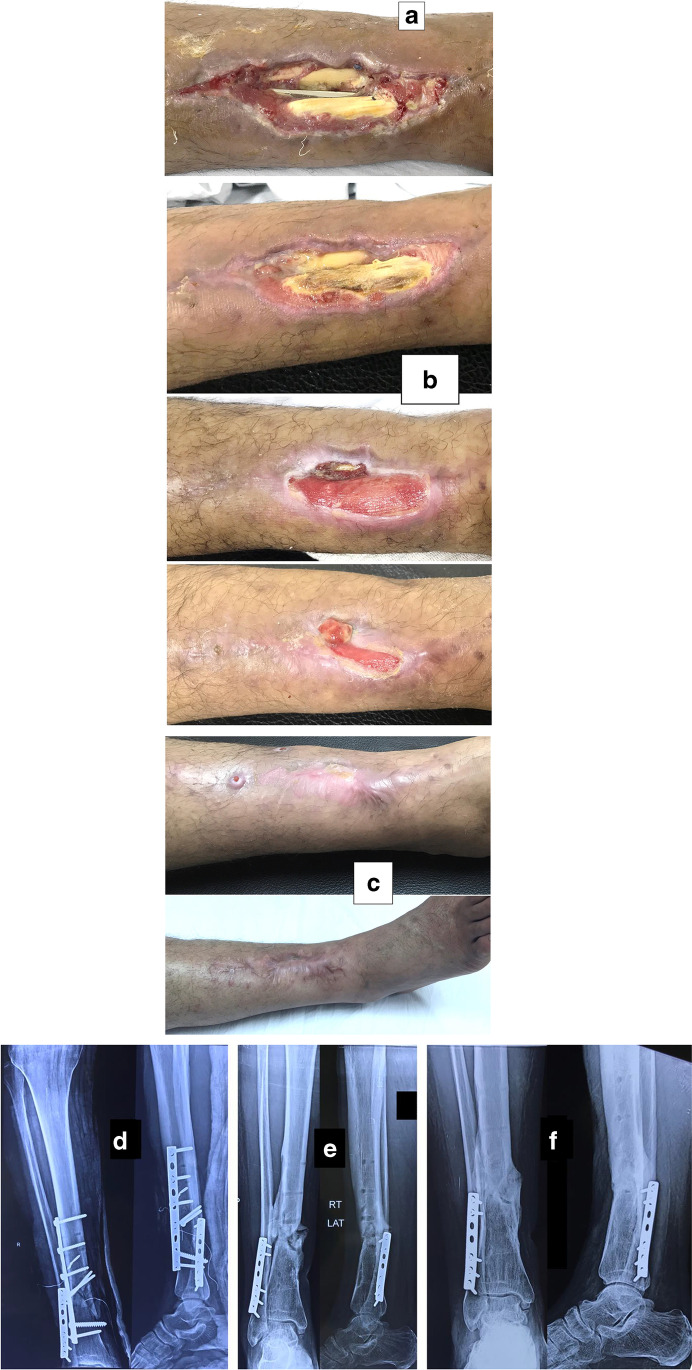
A 53-year-old diabetic smoker patient presented with a postoperative complicated infected wound in the distal part of his right leg following open reduction and internal fixation with plate and screws of closed distal tibial fracture. **a** Clinical photo at presentation with bone, plate and tendon exposure. **b** Clinical photos of different stages of wound repair. **c** Residual sinuses before implant removal (top) and complete bone coverage with dry scar after 39 months of follow-up (bottom). **d**, **e**, **f** Radiographs at presentation, after implant removal, and at the last follow-up

The original cause of the wound was open fracture in 34 cases (68%), infected tibial non-union in nine cases (18%), and seven cases (14%) of post-operative infection and wound dehiscence including four cases after fracture internal fixation (8%) and three Achilles’ tendon-related wounds (6%). The open fractures involved the proximal leg in four cases (8%), middle leg in 16 cases (32%), distal leg in eight cases (16%) and foot in six cases (12%). According to Gustilo and Anderson classification [11], open fractures were grade II in nine patients, grade IIIA in nine patients (Fig. 1) and grade IIIB in 16 patients (Fig. 2). Tibial non-union was distal in six cases (12%), middle tibial in two cases (4%) and proximal in one case (2%). The three patients with open Achilles’ tendon injuries included two patients (4%) with infected repair and one patient (2%) suffered postoperative infection and wound sloughing with tendon exposure after repair. Besides this case, exposed tendon was present in another two cases (Figs. 1, 3). Bone exposure was present in two cases. The first case was an open grade IIIB mid-tibial fracture with a failed soft tissue rotational flap reconstruction that was treated by negative pressure wound therapy for two weeks. The wound was large with an anteromedial bone exposure (Fig. 2). The second had exposed part of the anterolateral tibial surface after plate and screws fixation of a distal tibial fracture (Fig. 3). There were 21 smokers (42%), and six patients were diabetics (12%). Malnutrition, alcoholism and other causes of immunosuppression were not present in any patient of the study.

Along with wound care, the primary cause was handled individually. Open fractures were treated by debridement and fixation by either monolateral fixator or Ilizarov fixator. Infected non-unions were treated by debridement and Ilizarov external fixator. Regarding cases of post-operative infection after internal fixation, three cases had loose tibial plate treated by implant removal and Ilizarov fixator. The remaining case had a stable plate and screws fixation, and the implants were kept till sound tibial union (Fig. 3).

The descriptive statistics were done by IBM SPSS Statistics for Windows, Version 22.0 (IBM Corp., Armonk, NY, USA).

### The dressing technique

This was done after meticulous debridement and treatment of the cause (which was done by the authors), optimising the patient’s general condition, blood sugar control in diabetics and strict instructions on smoking cessation. An informed consent was obtained from all patients included in the study. As medical grade honey preparations are not available, patients brought original honey from local beekeepers without irradiation and kept it in dark containers at room temperature. The dressings were handled by the patient or one of the relatives at home after teaching them the steps of dressing during their initial hospitalisation where it was done by the resident. The dressing started by thorough wound washing with saline and using gauze to remove any superficial debris. No antiseptic was used in the study. After drying the wound, a ribbon of gauze soaked with honey was applied and folded into at least three layers. We used gauze to function as a mesh keeping honey to prolong its contact with the wound. The gauze length and amount of honey varied according to the wound size for covering the whole wound, filling its depth and hanging over its edges. A dressing was applied over the gauze and a crepe bandage was applied lightly. The frequency of dressing was variable according to the soaking of the dressing with exudates. This was done twice daily or once daily. With improvement of the wound condition, dressings were changed every other day. According to the culture results in infected cases, systemic antibiotics were given for four weeks.

### Assessment and follow-up

The patients were followed up weekly till sound wound healing, and monthly till treatment of original cause. On follow-up in the outpatient clinic, the patient or the relative was asked to perform the dressing in front of one of the authors to ensure the dressing consistency. In addition to radiographic follow-up of the orthopaedic condition, the parameters of wound follow-up were wound size and depth, clearance of discharge, progression of granulation tissue formation and epithelialisation, the condition of the adjacent skin (dermatitis, maceration, desiccation, oedema or excoriation), any adverse events, and time to complete wound healing. Photographs were taken for documentation. It was too difficult to precisely calculate the surface area of the wounds because of the highly variable irregularities of wound shapes. The size follow-up was done by monitoring the changes in the maximum wound length longitudinally and horizontally.

## Results

The orthopaedic condition of cases was followed up for a mean of 33.46 (range 22–47; SD 6.61) months. Wound sizes were variable. After debridement, skin closure with wide interrupted sutures was possible in ten open fractures and seven infected non-unions, and two open Achilles’ tendon-related wounds. Other open fracture wounds ranged from 8 to 20 cm longitudinally and 3 to 17 cm horizontally. Post-operative cases and two non-union wounds ranged from 10 to 13 cm longitudinally and 3 to 5 cm horizontally. Besides the 16 cases of infected non-unions and post-operative complications, 13 cases of open fractures were complicated by deep infection that necessitated debridement. Deep samples were taken during debridement from these 29 cases (58%) for culture and antibiotic sensitivity testing. *Staphylococcus aureus* was found in 12 cases (24%). Other wounds were infected by *Staphylococcus epidermidis* in seven cases (14%), *Klebsiella pneumonia* in four cases (8%), *Pseudomonas aeruginosa* in four cases (8%) and methicillin-resistant *Staphylococcus aureus* (MRSA) in two cases (4%). All cases showed improvement in all parameters of follow-up with complete wound healing and full coverage of bone (except a small part in one case) and tendons. There was improvement in the colour and progression of granulation tissue formation with gradual clearance of discharges. Contraction of the epithelialised tissue reduced the scar size. The surrounding skin remained healthy. The duration of complete wound healing since starting honey dressing ranged from four to 15 weeks according to the wound size.

Recurrence of deep infection occurred in three cases and treated by debridement with eventual control of infection. Regarding bone exposure cases, the first case showed healing of the wound with bone coverage except a small sequestered anteromedial partial cortical fragment that was excised with final dry bone coverage (Fig. 2). The second case with anterolateral distal tibial wound showed complete wound healing. However, the persistence of two small sinuses necessitated removal of the distal tibial plate and screws. The fracture was united, and the wound eventually became dry (Fig. 3). Exposed tendon cases showed necrosis of the tendon surface which was treated by superficial debridement in the clinic (Figs. 1, 3). Apart from these events, no remarkable side effects were recorded apart from mild itching sensation at the start of the dressing in five patients that was resolved after few days. There were no allergic reactions, secondary infections or surrounding skin complications. All patients were satisfied regarding their outcome with honey dressing.

## Discussion

For successful wound treatment, wound care should be combined with adequate management of the main cause and any systemic infection in addition to optimising the general condition of the patient [12, 13]. These principles were followed in the present study. Therefore, honey dressing is not a substitute for debridement, sequestrectomy or treating other causative conditions. It is a complementary care particularly in developing countries with limited resources. The rationale for using honey in this study was the different studies confirming its positive effects on wound healing as well as its antimicrobial and antioxidant actions. Moreover, different clinical studies showed good results with different types of wounds. A systematic review of 26 randomised and quasi-randomised controlled trials with 3011 patients having acute and different chronic wounds found that honey resulted in faster healing of both partial thickness burns compared to conventional treatment and infected post-operative wounds compared to antiseptics and gauze [10].

Published articles about the potential effects of honey dressings on orthopaedic-related wounds are still scarce. Lazarides et al. [14] used honey-impregnated dressings for ring fixator pin site care in 19 patients. Only two patients had superficial infection. Upadhyay et al. [15] evaluated the effect of honey dressing on 20 patients with traumatic orthopaedic wounds. Honey dressing only achieved excellent results in 12 cases. Four cases required multiple debridement and four patients needed local flap and skin grafting.

Besides promoting wound healing, the broad-spectrum antimicrobial effect of honey was attributed to several factors including hydrogen peroxide production, acidity, non-peroxide molecules (e.g. methyl syringate, defensin 1 and methylglyoxal) and osmotic activity [6, 16–19]. When mixed with wound exudates, honey produces low level of hydrogen peroxide which is bactericidal causing irreversible damage to the bacterial membranes, proteins, enzymes and DNA [3, 6]. Wounds show a biphasic response to hydrogen peroxide application. Low hydrogen peroxide concentrations enhance wound healing, but high levels delay healing [5, 6]. Antioxidants in honey protect the wounds from oxygen radicals which may result from hydrogen peroxide actions [5]. Honey pH is between 3.2 and 4.5, and this acidity inhibits bacterial growth as the optimal pH of most micro-organisms is about 7.2 to 7.4 [20]. Moreover, this acidity promotes wound healing through increasing the oxygen release from haemoglobin [21].

The hyperosmolarity of honey dehydrate bacteria thus preventing their proliferation or killing them [3, 19, 22]. Additionally, this osmotic power draws lymphatic fluid from the wound bed and consequently enhancing the lymph outflow as in negative pressure wound therapy [21, 23]. This moist environment is necessary to remove damaged, dead and infected tissues. Besides hyperosmolarity, this autolytic painless debridement is attributed to the presence of the protease enzyme [3].

Owing to these combined actions targeting multiple and different sites in fighting bacteria, honey is effective against a broad spectrum of pathogens, including resistant bacteria and fungi [4, 22, 24]. Honey is effective against *Staphylococcus aureus*, *Escherichia coli*, *Pseudomonas aeruginosa*, Acinetobacter and Stenotrophomonas, MRSA, vancomycin-resistant Enterococcus and extended-spectrum beta-lactamase (ESBL)-producing strains of *Escherichia coli*, Klebsiella species and Enterobacter species [3, 21, 25]. A systematic review analysed 16 articles that included 18 different honey types against 32 variable bacterial species, including numerous multidrug-resistant (MDR) strains. All honey types demonstrated a high efficacy against the tested bacterial species, including MDR strains [26]. The minimum inhibitory concentrations (MIC) values of honey were reported to be less than 11%. Therefore, even when honey diluted by the exudate, it still has potent antibacterial activity [21].

Regarding systemic antibiotics, the optimal duration of therapy for treating traumatic and implant-related orthopaedic infections remains unclear [27]. It is largely dependent on expert opinion [28]. Rod-Fleury et al. [29] in a study of 49 adults with implant-free chronic osteomyelitis found that a post-debridement antibiotic administration more than 6 weeks did not show increased remission incidences. Benkabouche et al. [30] performed a randomised trial with 123 cases of implant-related osteomyelitis and reported that four weeks duration of antibiotic treatment was not inferior to the currently recommended six weeks after implant removal.

The current study presented chronic wounds of different aetiologies. A wound is considered chronic if its healing is delayed after two to eight weeks. Chronic wound infections are challenging due to the biofilm formation that provide bacterial resistance to antibiotics by extracellular barrier of polymeric substances [25, 31]. Honey is effective in reducing the biofilm of both drug-sensible and drug-resistant strains of Gram-positive and Gram-negative bacteria [18, 22, 31, 32]. Differential gene expression analysis proved the honey ability for downregulation of several genes related to biofilm formation [22, 32].

Bacterial resistance to honey has not been reported because of the multiple antimicrobial mechanisms and components working additively and/or synergistically [6, 18, 21, 22, 24, 25]. This multifactorial nature of honey could explain the wound healing progression in our study despite discontinuation of the initial course of antibiotics administered before complete epithelialisation without any secondary infections.

Medical grade honey (MGH) is honey that has been sterilised by gamma irradiation. It is available in the form of honey in tubes, impregnated dressings and gels for wound care [25]. Manuka honey is the most often used one as it was the first MGH extensively investigated [18]. However, every honey also has antibacterial actions as demonstrated in variable studies from different geographical locations [18, 22, 26]. MGH was not used in our study as it is unavailable. Secondly, there are no reports of infection after using non-irradiated honey [5, 22]. Thirdly, several studies demonstrated the efficacy of using honey without irradiation [12, 13, 15, 33–36]. Lastly, the local honey effectiveness was presented in different in vitro [32, 37] and clinical studies [13, 33, 36].

The present study demonstrated the efficacy of honey on healing of different orthopaedic-related wounds. This dressing method was easy, non-sticky to the wound bed, available and with low cost. The usefulness was demonstrated even with bone and tendon exposure. Undoubtedly, wounds with exposed tendons or bones are serious complications as they are often associated with an increased risk of adverse outcome. This efficacy of honey with exposed tendon and bone could be secondary to the moist hyperosmolar environment and the protective barrier effect which prevent these structures from desiccation in addition to the multifactorial antimicrobial actions. Two single case reports were published and showed the effectiveness and safety after honey dressing. Teobaldi et al. [23] treated a chronic posterior leg ulcer in a diabetic patient with exposed tendon and reported complete epithelisation of more than half of the ulcer after eight weeks and complete tendon coverage after 18 weeks. Astrada et al. [12] presented a diabetic foot ulcer with exposed bone and achieved complete re-epithelialisation after two months of honey dressing. While these reports did not mention any tendon complication, we observed only superficial tendon necrosis. However, the integrity of the remaining most of the tendon persisted.

The current study has the limitations of a relatively small number of cases, lacking controls, heterogenicity of wounds and the inability to ensure the consistency of honey. Power analysis was not done to assess the sample size prior to study performance. As a result, we do not have statistical power to draw statistically significant conclusions. The cost was not compared to other types of dressings. Performing a double-blinded controlled study on honey dressing is technically difficult due to its characteristic physical properties and odour [38]. The heterogenicity is due to the variable aetiologies and it is too difficult to have a series of patients with the same wound site, size and depth. The authors wanted to assess the effect of honey on different wounds. Moreover, there is lack of validated method of wound evaluation. This is because there is no consensus on the most appropriate parameters of wound healing [39]. However, the presented multiple follow-up parameters could compensate for this limitation. Superficial wound swabs for bacterial culture were not used in the study because they often reflect contamination, are prone to false results and may lead to unnecessary antimicrobial treatment [40, 41]. Randomised controlled large-scale studies are recommended to provide a better insight on evaluation of this method of orthopaedic wound care.

## Conclusions

With treating the underlying aetiology and optimising the patient’s general condition, honey was an effective, simple and affordable method of infected wound care in different orthopaedic conditions even with exposed bone or tendons. For the proven biological and antibacterial activities, honey has the potential to be new therapeutic choice which should be considered in the clinical orthopaedic practice for infected wound care.

## Data Availability

The datasets generated during and/or analysed during the current study are available from the corresponding author on reasonable request.
